# Investigation of damping coefficients for elastic collision particles utilizing the acoustic frequency sampling method

**DOI:** 10.1038/s41598-024-57487-z

**Published:** 2024-04-20

**Authors:** Xinlin Shi, Wenzhen Zhong, Qingxin Zhao, Runzi Li, Dengchao Sun

**Affiliations:** School of Mechanical Engineering, University of Jinan, Jinan, 250022 China

**Keywords:** Particle damping coefficient, Acoustic frequency sampling, Discrete element method, Material density, Elastic modulus, Theoretical particle physics, Ceramics, Composites, Metals and alloys, Techniques and instrumentation, Computational methods

## Abstract

The damping coefficient serves to quantify the energy dissipation in particle collisions and constitutes a crucial parameter in discrete element simulations. Nevertheless, the factors influencing the damping coefficient remain unclear, and the damping coefficients of the majority of materials have not been precisely determined. In this investigation, the damping coefficients of eight representative particles were studied using the acoustic frequency sampling method, and the correlations between these coefficients and collision velocity, material density, and elastic modulus were analyzed. The findings indicate that damping coefficients exhibit insensitivity to velocity in strongly elastic and moderately elastic material particles. Conversely, for weakly elastic material particles, damping coefficients demonstrate an increase with rising velocity. The damping coefficient of metallic particles exhibits a linear relationship with material density and elastic modulus.

## Introduction

Granular materials, which consist of numerous discrete solid particles, are intricate systems characterized by multiple body interactions^1,2^. These materials are ubiquitous in natural settings, industrial construction, and agricultural production, encompassing various examples such as hailstones, pharmaceutical powders, iron-based powders, and agricultural crop seeds. Notably, granular materials display notable distinctions from both fluids and solids and possess distinctive properties. Consequently, researchers must employ appropriate methodologies and develop suitable models to facilitate comprehensive investigations in this field.

The discrete element method (DEM) is a numerical simulation technique that is utilized to examine the dynamics of particle systems. It offers a systematic depiction of particle motion and finds extensive applications in the simulation analysis of granular materials^3–6^. Within the Discrete Element Method, the soft-sphere model^7,8^ is particularly suitable for simulating the dense flow of particles, thus resulting in broader applications^9–11^. The solution models of the soft-sphere model primarily consist of linear models, which are based on Hooke's law, and nonlinear models, which are based on Hertz theory. However, the determination of damping coefficients in the model is often based on subjective judgment, leading to uncertainties and randomness in the simulation outcomes. Consequently, researchers have undertaken a series of investigations on damping coefficient models and precise measurement methods^12,13^. Shäfer et al.^14^ examined particle collisions using the linear model and derived the relationship between the damping coefficient $\gamma$, restitution coefficient *e*_*n*_, and collision duration *t*_*n*_.

$$ e_{n} = \exp \left( { - \frac{{\gamma_{n} }}{{2m_{eff} }}t_{n} } \right). $$

In this equation, *m*_*eff*_ represents the equivalent mass, and *k*_*n*_ denotes the spring stiffness coefficient.

In their study, Zhong et al.^15^ utilized a linear elastic-damping model and employed the acoustic frequency sampling method to calibrate the damping coefficient of the steel sphere. They established a correlation between the damping coefficient $\gamma$ and the energy attenuation coefficient $\eta$ for collision events.

$$ \eta = \frac{2\gamma }{{1 + \gamma }}. $$

By utilizing the calibrated damping coefficient, they successfully simulated the process of particle stacking and obtained results that aligned with well-established experimental findings. This investigation effectively demonstrated the viability of employing the acoustic frequency sampling method for measuring the damping coefficient. Subsequently, Zhou et al.^16^ conducted steel ball-collision-rebound experiments using the acoustic frequency sampling method and complemented their findings with Discrete Element Method simulations. The outcomes of their research revealed that the normal viscous damping ratio of the steel sphere was determined to be 0.16. It was also observed that a higher normal viscous damping ratio correlated with greater energy absorption during collisions, resulting in lower rebound heights of the particles. In the realm of vibration impact research, Hunt and Grossley^17^ have put forth a nonlinear model that addresses the damping coefficient $\gamma$ and the restitution coefficient *e*_*n*_.

$$ \gamma = \frac{3}{4}\left( {1 - e_{n} } \right). $$

A novel mathematical relationship was derived by Hu et al.^18^ through numerical analysis, specifically fitting restitution coefficients within the range of 0.05 to 1.0.

$$ \gamma = \frac{1}{2}\ln_{{e_{n} }} \frac{a}{{b + \ln_{{e_{n} }} }}. $$

Subsequently, Caserta et al.^19^ extended the range of restitution coefficients from 0.3978 to 1.0 by introducing parameter *c*. This expansion allowed for further improvements in the nonlinear contact model of the damping coefficient.

$$ \gamma = \frac{1}{2}\ln_{{e_{n} }} \frac{a}{{b + c\ln_{{e_{n} }} }}. $$

The values of *a*, *b*, and *c* were determined through numerical nonlinear fitting techniques.

Furthermore, the inclusion of granular materials with specific damping properties plays a crucial role in collision dampers as it significantly impacts the shock absorption capabilities of said dampers. Li^20^ and Ye^21^ conducted a thorough examination of the damping characteristics of zinc powder and copper powder particles. The findings revealed that dampers incorporating these two types of particles showcased commendable performance in mitigating vibrations. Notably, the damping coefficient serves as a vital parameter in quantifying the damping of granular materials. This study, therefore, assesses the damping characteristics of the aforementioned material utilizing measuring its respective damping coefficient.

Precisely assessing the damping coefficient holds immense importance, be it in discrete element simulations or the enhancement of damping function in materials. Nevertheless, the precise determinants that affect the damping coefficient remain elusive, necessitating accurate determination of the damping coefficients for most materials. Consequently, in this investigation, the damping coefficients of eight representative particles, encompassing both polymer and metal materials, were ascertained by employing a high-precision acoustic frequency sampling method. The investigation analyzes the characteristics of collision acoustic frequency waveform patterns for diverse materials and elucidates the impacts of crucial factors, including velocity, particle density, and elastic modulus, on the damping coefficients.

## Materials and methods

### Materials

Eight types of particles were chosen for the experiment, encompassing both common polymer and metal particles. Among these, the polymer particles comprise PP (polypropylene), POM (polyoxymethylene), and acrylic; the metal particles consist of 304 stainless steel, TA2 titanium, H62 brass, aluminum, and tungsten steel (YG6). To maintain consistency, the particles have an approximate diameter of 15 mm, and the specific parameters are detailed in Table 1.

**Table 1:** Experimental material parameters.

Materials	Name	Density	Elastic modulus	Diameter	Note
		g·cm^–3^	GPa	mm	
Polymer	PP (polypropylene)	0.89	1.4	15.01	
	Acrylic	1.17	1.35	15.10	
	POM (polyoxymethylene)	1.41	2.6	14.80	
Metals	Stainless steel (304)	7.93	200	14.99	18% Cr, 8% Ni
	Brass (H62)	8.44	100	15.00	60.5–63.5% Cu
	Titanium (TA2)	4.50	110	15.10	
	Aluminum	2.71	70	15.01	
	Tungsten steel (YG6)	14.92	400	14.98	WC:94%, Co:6%

### Damping coefficient measurement experimental apparatus

**Figure 1:**
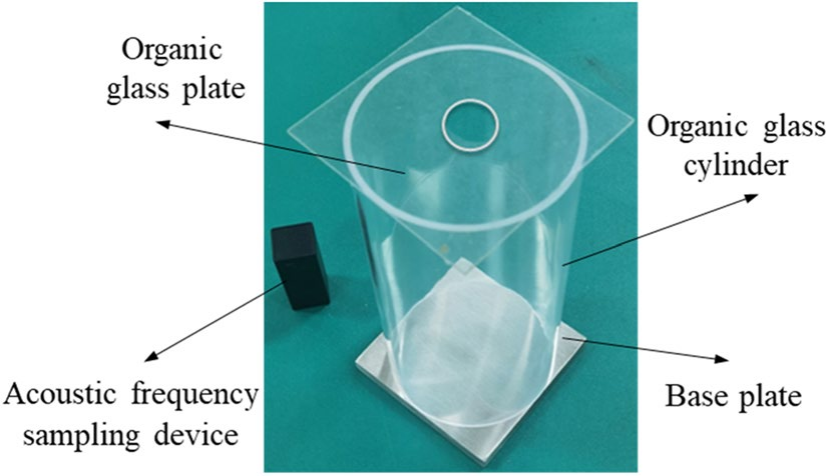
Damping coefficient measurement experimental apparatus.

In this investigation, a test apparatus for determining damping coefficients utilizing the acoustic frequency sampling method was designed and constructed, as depicted in Fig. 1. The apparatus comprises four components: a plexiglass flat plate with a central hole, a plexiglass cylinder, a base plate, and an acoustic frequency sampling device. The Plexiglas cylinder has a height (*H*) of 200* mm* and a diameter (*Φ*) of 100* mm*, with *H* determining the initial falling height of various particles. The base plate, with dimensions of 150* mm* × 150* mm* × 10* mm*, is composed of the same material as the test sphere. Its thickness and support strength are designed to prevent deformation during collisions. The sampling frequency for acoustic frequency is set at 48* kHz*. The central aperture in the Plexiglas plate determines the particles’ descent position. The combined height (*H*) of the Plexiglas cylinder and the central hole in the Plexiglas guarantees uniform initial conditions for diverse particles.

**Figure 2:**
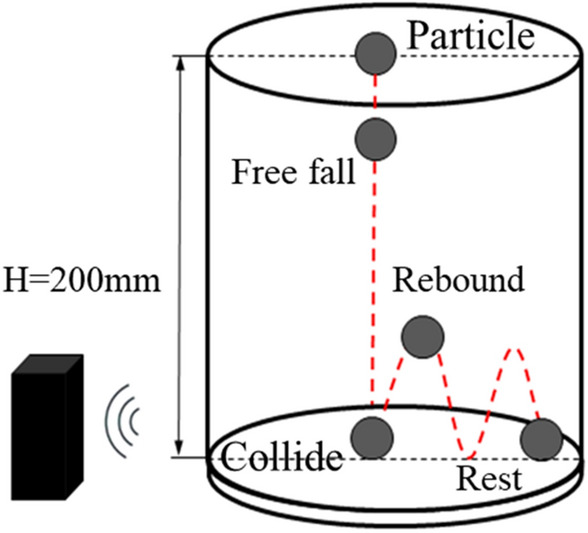
Schematic diagram of particle collision process.

**Figure 3:**
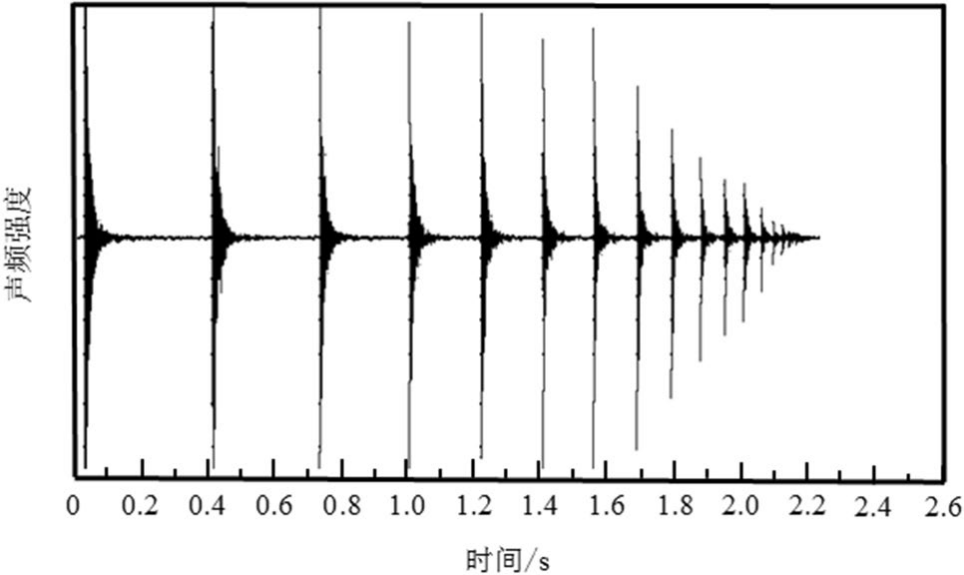
Acoustic frequency waveform diagram of particle collisions.

The experimental procedure employed to determine the damping coefficient is illustrated in Fig. 2. A particle, initially at rest, is released from the central aperture of the organic glass plate and descends freely until it collides with the base plate. Upon impact, the particle instantaneously bounces back and ascends to its maximum height before commencing the subsequent cycle. This process is recurrently executed until the particle eventually comes to a complete halt. The acoustic frequency sampling device automatically captures the acoustic information generated by each collision. Subsequently, the captured acoustic data is subjected to processing using acoustic analysis software, resulting in the acquisition of the acoustic waveform diagram depicted in Fig. 3. The waveform diagram in Fig. 3 documents the entire progression of particle collisions, from the initial stage to the final stage. Temporal representation is delineated along the horizontal axis, while the vertical axis corresponds to the intensity of the acoustic frequency. Each pulse depicted in the waveform diagram signifies a collision event. Notably, the early pulses in the acoustic waveform diagram exhibit higher intensity, whereas the subsequent pulses demonstrate a rapid decrease in intensity. This observation aligns with the physical phenomenon of high-intensity collisions occurring during the early stage, followed by low-intensity collisions in the later stage. Consequently, the acquired acoustic waveform diagram attests to the reliability of the experimental results.

### Determination of the damping coefficient

The temporal dimension on the x-axis is depicted in Fig. 3, which accurately denotes the duration between two successive pulses. This duration effectively measures the time interval corresponding to two distinct instances of free fall. By utilizing this time interval, it becomes possible to compute the kinetic energy of the particles. The calculation methodology is as follows: Assuming the temporal occurrence of the *n*th collision is denoted as *t*_*n*_, following the equation governing kinetic energy, the kinetic energy preceding the *n*th collision is given by,

1 $$ E_{t,n - 1} = \frac{1}{2}m\left( {g\frac{{t_{n} - t_{n - 1} }}{2}} \right)^{2} , $$

and the kinetic energy after the *n*th collision can be expressed as,

2 $$ E_{t,n - 1} = \frac{1}{2}m\left( {g\frac{{t_{n + 1} - t_{n} }}{2}} \right)^{2} . $$

Introducing the energy attenuation coefficient for the *n*th collision as,

3 $$ \eta = \frac{{E_{t,n - 1} - E_{t,n} }}{{E_{t,n - 1} }} = \frac{{\left( {t_{n} - t_{n - 1} } \right)^{2} - \left( {t_{n + 1} - t_{n} } \right)^{2} }}{{\left( {t_{n} - t_{n - 1} } \right)^{2} }}, $$

and considering the time interval Δ*t*_*n*_ = *t*_*n*+*1*_-*t*_*n*_ between the *n*th collision and the (*n* + *1*)th collision, we can derive the relationship.

4 $$ \eta = \frac{{\Delta t_{n - 1}^{2} - \Delta t_{n}^{2} }}{{\Delta t_{n - 1}^{2} }}. $$

Hence, through the quantification of the temporal gap between successive collisions, it becomes feasible to compute the energy attenuation coefficient associated with a particular collision.

In the entirety of the process, where the particles descend vertically, the presence of frictional forces can be disregarded. Consequently, the only factor influencing the decrease in energy during particle collisions is damping. As a result, we establish a correlation between the coefficient of kinetic energy attenuation and the damping coefficient. By utilizing the relationship between the damping coefficient and the coefficient of kinetic energy attenuation, as provided in reference^14^, it is possible to ascertain the damping coefficient of particles during collision processes.

5 $$ \gamma = \frac{\eta }{2 - \eta } = \frac{{\Delta t_{n - 1}^{2} - \Delta t_{n}^{2} }}{{\Delta t_{n - 1}^{2} + \Delta t_{n}^{2} }}. $$

To uphold the trustworthiness of the calculation outcomes, it is imperative to derive the damping coefficient of the particles by averaging multiple damping coefficients. By assuming a series of high-intensity collisions among the particles, the average value is determined as follows:

6 $$ \gamma = \frac{1}{k}\sum\limits_{n = 1}^{k} {\left( {\frac{\eta }{2 - \eta }} \right)} = \frac{1}{k}\sum\limits_{n = 1}^{k} {\left( {\frac{{\Delta t_{n - 1}^{2} - \Delta t_{n}^{2} }}{{\Delta t_{n - 1}^{2} + \Delta t_{n}^{2} }}} \right)} . $$

## Results and discussion

### Acoustic frequency waveform of particles

**Figure 4:**
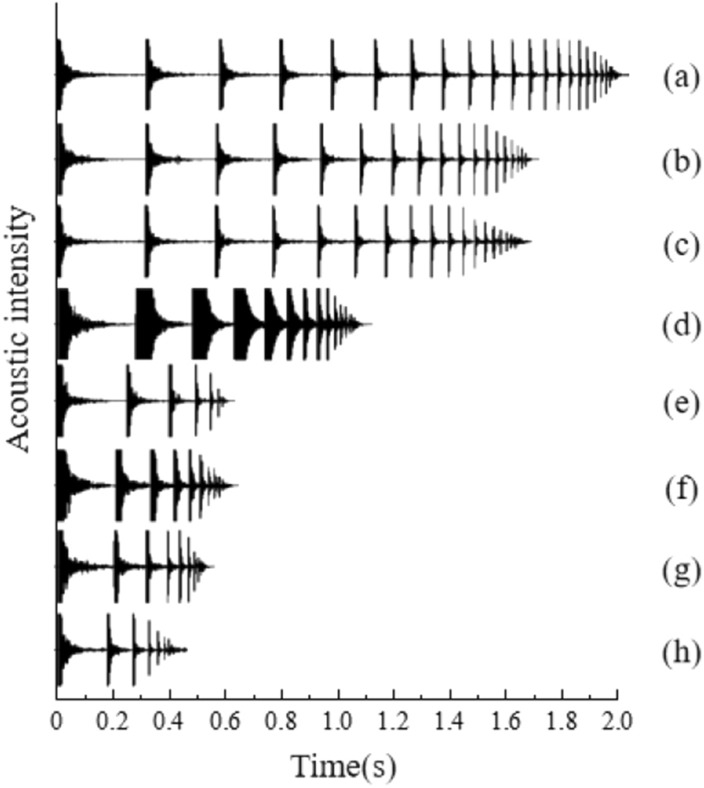
Acoustic frequency waveforms of particles of various materials. (**a**) POM; (**b**) Acrylic; (**c**) PP; (**d**) Tungsten steel; (**e**) Titanium; (**f**) Stainless steel; (**g**) Brass; (**h**) Aluminum.

Acoustic frequency waveforms of particles with various materials were acquired through acoustic frequency analysis software, illustrated in Fig. 4. Through a comparison of the acoustic frequency waveform graphs for each material, the eight materials were classified as strong elasticity, medium elasticity, and weak elasticity. Materials exhibiting strong elasticity encompass (a) POM, (b) acrylic, and (c) PP; those with moderate elasticity include (d) tungsten steel; and materials with weak elasticity comprise (e) titanium, (f) stainless steel, (g) brass, and (h) aluminum.

Materials with strong elasticity undergo a high frequency of collisions (20 or more), involving numerous high-intensity collisions followed by low-intensity collisions, leading to extended collision durations (exceeding 2 s); conversely, materials with moderate elasticity undergo fewer collisions (around 12) and have shorter collision durations (approximately 1.2 s). Following several high-intensity collisions, materials with moderate elasticity cease colliding after a reduced number of subsequent low-intensity collisions. In contrast, materials with weak elasticity cease colliding after a minimal number of collisions (only 8) and a brief duration (0.6 s). The aforementioned analysis indicates that materials with strong elasticity exhibit a high collision frequency and prolonged collision durations, materials with weak elasticity showcase a low collision frequency and abbreviated collision durations, and materials with moderate elasticity fall in between.

Through qualitative analysis of the acoustic frequency waveform diagram, it can be observed that materials with high elasticity demonstrate a reduced level of energy loss and a more gradual rate of energy dissipation during a singular collision. Conversely, materials with weak elasticity experience a higher degree of energy loss and a quicker rate of energy dissipation. Medium-elastic materials exhibit an energy dissipation that falls within the spectrum of these two extremes.

### Damping coefficient of particles

**Figure 5:**
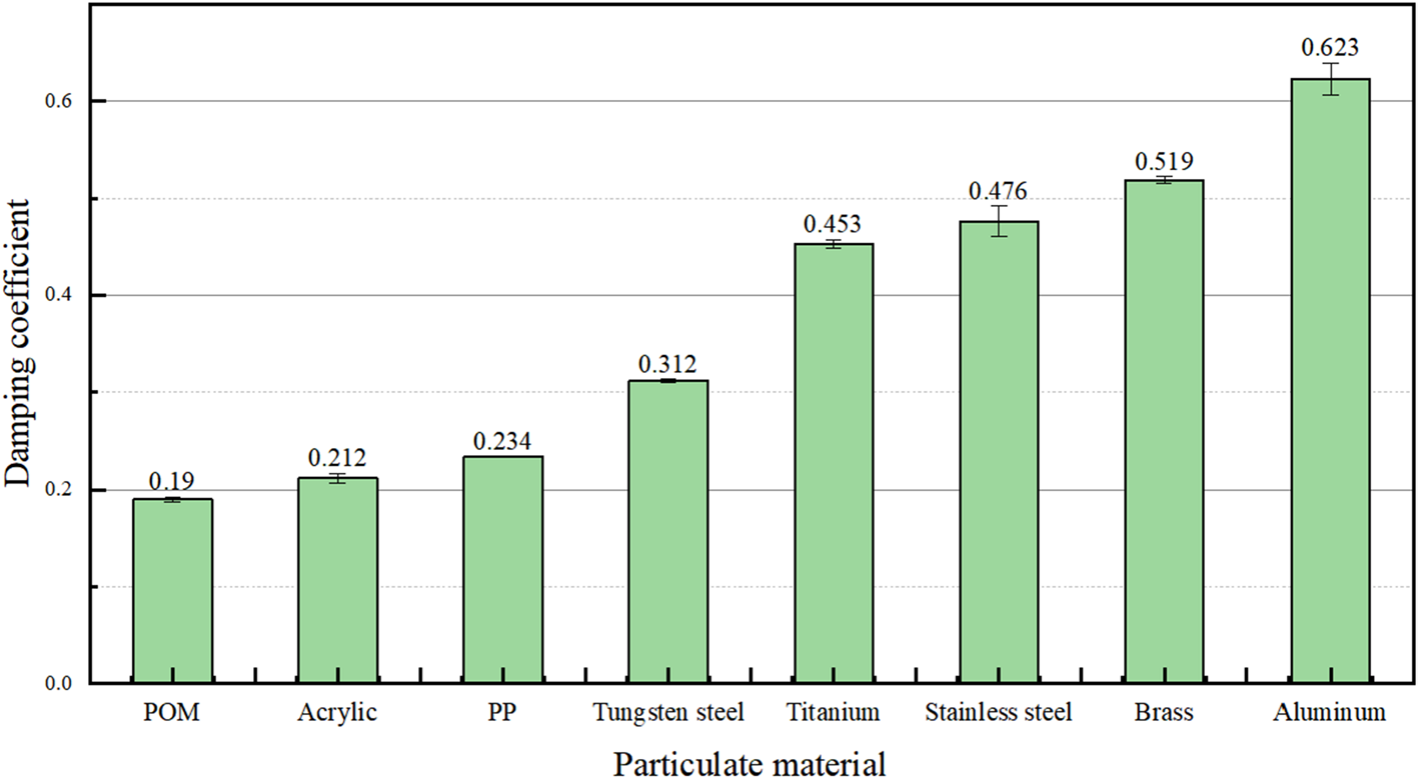
Damping coefficient of different material particles.

To precisely ascertain the damping coefficients of particles, the acoustic frequency data from the high-intensity collision phase in the acoustic frequency waveform of each material's particles were chosen, as this period corresponds to the primary energy dissipation of the particles. Kinetic energy attenuation coefficients for each particle during three repeated collision tests were computed using Eq. (4). Subsequently, the damping coefficients for each particle were derived using Eq. (6), and the resulting coefficients for particles composed of various materials are depicted in Fig. 5. The damping coefficients for stainless steel particles and brass particles align with findings from related studies.

Examining the damping coefficients of particles from various materials reveals that those of strong elastic materials (acrylic, POM, and PP) are low. As polymers, they exhibit the characteristic of minimal energy loss in individual collisions and a gradual loss rate. Conversely, the damping coefficients of weakly elastic materials (titanium, stainless steel, brass, and aluminum) fall within the range of 0.4 to 0.7, with a significant portion of energy dissipation attributed to damping. The damping coefficient for particles of moderately elastic material (tungsten steel) is 0.312, suggesting that some energy is lost due to damping. Discrepancies exist in the damping coefficients of particles from different materials, potentially linked to specific factors or the inherent properties of the materials. Further investigation and analysis are warranted.

#### Relationship between damping coefficient and collision velocity

**Figure 6:**
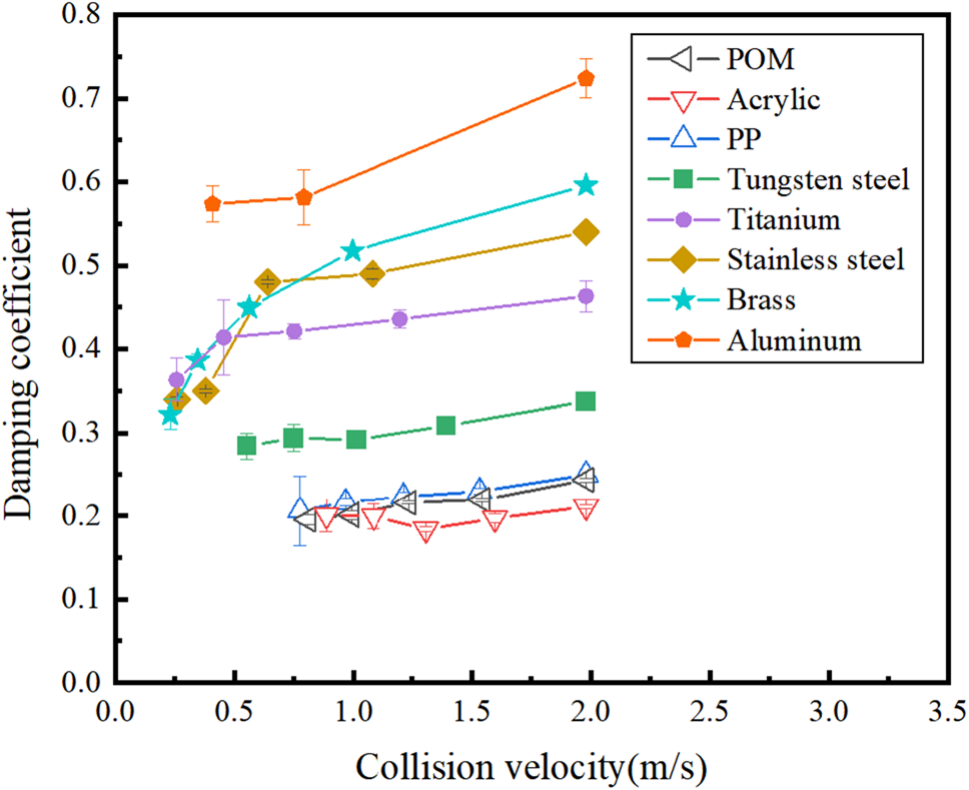
Variation of particle damping coefficient with velocity.

Examining the potential impact of velocity changes on damping coefficients is a scientifically significant question, thus prompting an investigation into the relationship between the damping coefficients of particles during each collision and their collision velocities. Damping coefficients for particles made of strongly elastic and moderately elastic materials are small and exhibit minimal variation with increasing velocity, displaying an approximately horizontal trend. This suggests that velocity has an insignificant impact on the damping coefficients for strongly elastic materials, as depicted in Fig. 6. Damping coefficients are more sensitive to velocity changes, and all exhibit an increasing trend with rising velocity. In the case of particles from weakly elastic material (aluminum), their damping coefficients also exhibit an inclination to rise with increasing velocity. Notably, among them, the damping coefficient of aluminum particles with the highest damping coefficient demonstrates the most pronounced trend with velocity.

In conclusion, speed changes have minimal impact on the damping coefficients of strong and moderately elastic materials. Conversely, the damping coefficient of weakly elastic materials rises with increasing speed. Upon analysis, it is evident that strong elastic materials, being polymers with low density and lightweight, exhibit strong elasticity. The uniform energy loss in each collision renders them almost impervious to changes in speed. Conversely, weakly elastic materials experience greater energy loss due to damping during collisions, resulting in a rapid decrease in rebound height over a short duration. Consequently, they are considerably influenced by changes in speed.

#### Relationship between damping coefficient and density

**Figure 7:**
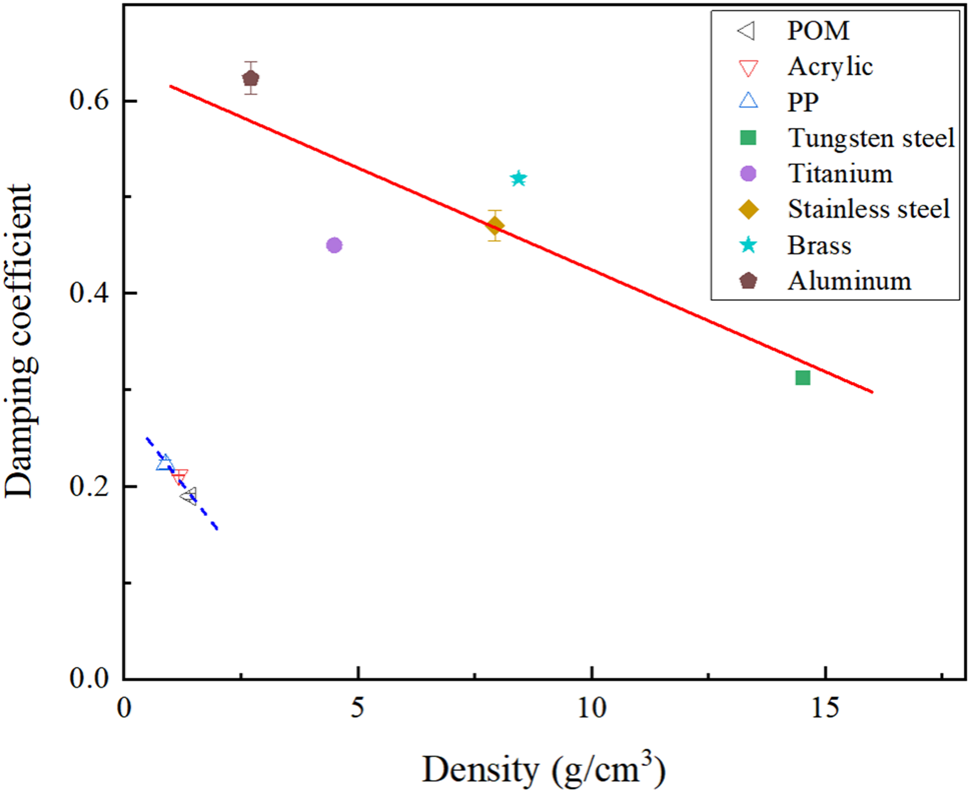
Variation of particle damping coefficient with density.

The correlation between particle damping coefficient and density is illustrated in Fig. 7. Observing the figure, it is evident that the particle damping coefficient decreases with an increase in density for both polymer and metallic materials. Notably, for metallic material particles with substantial variations in density and damping coefficient, a distinct linear relationship between the damping coefficient and density is evident. However, in the case of polymer materials with comparable damping coefficients and densities (POM, acrylic, PP), the linear relationship between damping coefficients and densities is less apparent. This outcome can be rationalized by the observation that metallic materials with lower density exhibit a larger damping coefficient. This is attributed to the faster movement of atoms or molecules within these materials, enabling them to absorb more energy and consequently leading to higher damping energy loss. The equation expressing the relationship between density and the damping coefficient of metallic material was derived from the fitted curve.

7 $$ \gamma = - 0.021\rho + 0.636. $$

#### Relationship between damping coefficient and elastic modulus

**Figure 8:**
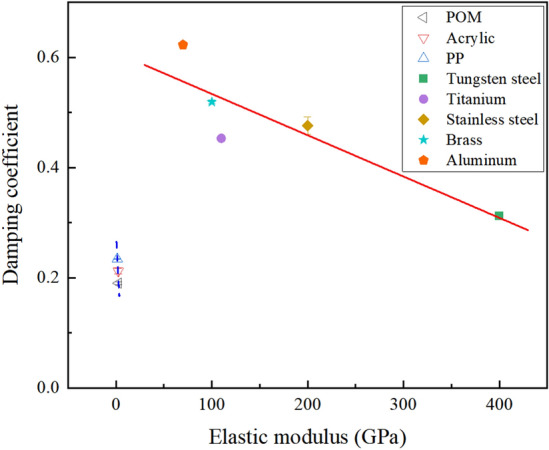
Variation of particle damping coefficient with elastic modulus.

The modulus of elasticity is a crucial metric for assessing a material's resistance to elastic deformation. To delve deeper into the factors influencing the material damping coefficient, the connection between the particle damping coefficient and variations in the specific material's elastic modulus was examined. Figure 8 illustrates that polymer materials, characterized by a small elastic modulus and a maximum difference of only 2, demonstrate a weak linear correlation between their damping coefficient and elastic modulus. Conversely, the relationship between the damping coefficient and elastic modulus for metallic materials demonstrates a linear pattern akin to that observed with density. The linear fitting equation is as follows:

8 $$ \gamma = - 0.00075E + 0.2609. $$

Metallic materials with a lower modulus of elasticity typically exhibit enhanced deformation ability, enabling them to absorb and dissipate external energy through more extensive deformation during collisions. Therefore, the damping coefficient increases as the modulus of elasticity decreases.

## Conclusions

In this study, the damping coefficients of eight types of particles made of polymer and metal were measured using the acoustic frequency sampling method. The corresponding acoustic frequency waveforms were also obtained. The damping coefficients of these granular materials were calculated. Based on these calculations, the study investigated the relationships between the velocity of granular motion, density, modulus of elasticity, and the damping coefficients. The following main conclusions were drawn:

Analysis of the material's acoustic frequency waveform reveals the following: strong elastic materials (acrylic, POM, and PP) experience a high frequency of collisions, long collision durations, and minimal energy loss per collision, resulting in a gradual energy dissipation; weakly elastic materials (titanium, stainless steel, brass, and aluminum) undergo a lower collision frequency, shorter collision durations, and substantial energy loss per collision, leading to rapid energy dissipation; materials with medium elasticity (tungsten steel) exhibit characteristics between the two extremes.

The damping coefficients of strongly elastic and moderately elastic materials remain nearly unaffected by changes in velocity. In contrast, the damping coefficients of weakly elastic materials exhibit significant variations with velocity, displaying an increasing trend with higher velocities. The damping coefficient of particles exhibits a linear correlation with the density and elastic modulus of the material. These studies highlight that the damping coefficient of a material is a complex physical quantity influenced by various factors.

## Data Availability

The authors confirm that the article is about the acoustic frequency waveforms of particles, the damping coefficients of particles, and that the article provides data to support these findings.
